# DNA Damage Response and Autophagy: A Meaningful Partnership

**DOI:** 10.3389/fgene.2016.00204

**Published:** 2016-11-21

**Authors:** Aristides G. Eliopoulos, Sophia Havaki, Vassilis G. Gorgoulis

**Affiliations:** ^1^Molecular and Cellular Biology Laboratory, Division of Basic Sciences, Medical School, University of CreteHeraklion, Greece; ^2^Institute of Molecular Biology and Biotechnology, Foundation for Research and Technology HellasHeraklion, Greece; ^3^Molecular Carcinogenesis Group, Department of Histology and Embryology, Medical School, National and Kapodistrian University of AthensAthens, Greece; ^4^Faculty Institute of Cancer Sciences, Manchester Academic Health Sciences Centre, University of ManchesterManchester, UK; ^5^Biomedical Research Foundation of the Academy of AthensAthens, Greece

**Keywords:** DNA damage, autophagy, mitophagy, evolution, senescence, repair, inflammation, cell death

## Abstract

Autophagy and the DNA damage response (DDR) are biological processes essential for cellular and organismal homeostasis. Herein, we summarize and discuss emerging evidence linking DDR to autophagy. We highlight published data suggesting that autophagy is activated by DNA damage and is required for several functional outcomes of DDR signaling, including repair of DNA lesions, senescence, cell death, and cytokine secretion. Uncovering the mechanisms by which autophagy and DDR are intertwined provides novel insight into the pathobiology of conditions associated with accumulation of DNA damage, including cancer and aging, and novel concepts for the development of improved therapeutic strategies against these pathologies.

## The DNA Damage Response

The term ‘DNA damage response’ (DDR) refers to a network of intracellular pathways that sense and resolve damaged DNA. If unrepaired, DNA lesions may result in cell death (Roos et al., 2016) but can also be a major source of genomic instability particularly when cell death pathways have been deactivated (Halazonetis et al., 2008). DDR signaling has been extensively reviewed elsewhere (Halazonetis et al., 2008; Jackson and Bartek, 2009; Marechal and Zou, 2013; Ribezzo et al., 2016); below we provide a summary of some of its components and their functions which are most relevant to this review.

**FIGURE 1:**
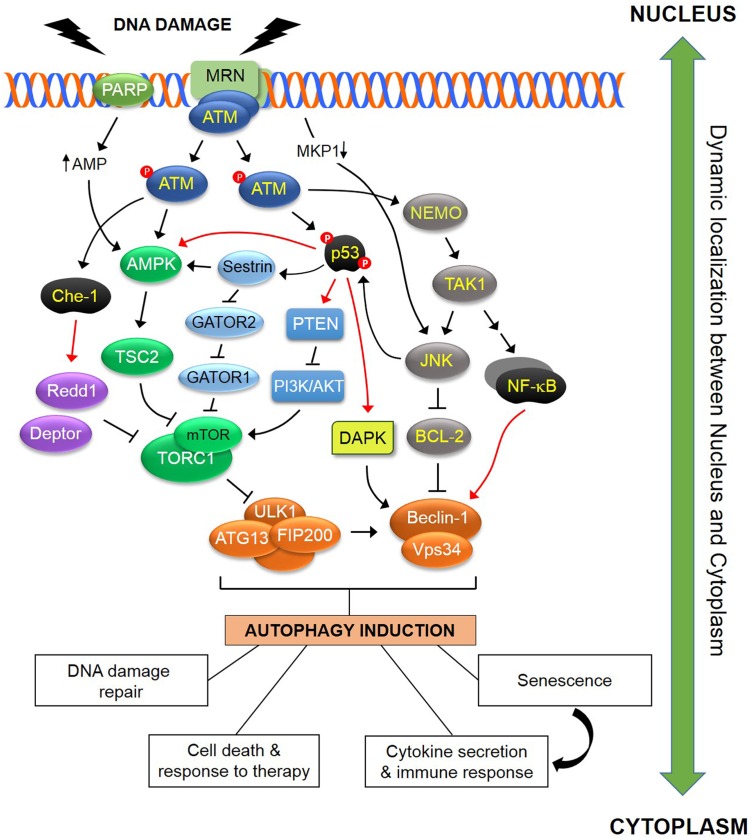
**Schematic representation of some of the known pathways linking DDR to autophagy.** In response to DNA damage, ATM is autophosphorylated within a MRN multiprotein complex that binds DSBs. Activated ATM initiates a pathway that results in activation of AMPK and its target TSC2 which functions as inhibitor of TORC1. As ULK1-dependent autophagosome formation is negatively regulated by TORC1, this ATM pathway induces autophagy. In addition, ATM directly phosphorylates and stabilizes p53 which transcriptionally regulates various regulators of the autophagic pathway including AMPK, DAPK and PTEN. Sestrins, which are regulated by p53, influence TORC1 activity in an AMPK-dependent and AMPK-independent manner, the latter through a GATOR2-GATOR1-RAGB/A signaling pathway (see text for details). The ATM-mediated phosphorylation and activation of the RNA polymerase II-binding protein Che-1, leads to increased transcription of two mTOR inhibitors, Redd1 and Deptor. ATM contributes to the activation of a NEMO-dependent TAK1-ATM-NEMO-NFκB pathway that transcriptionally modulates Beclin-1. TAK1 may also mediate activation of JNK which in turn phosphorylates Beclin-1 and releases it from the inhibitory effect of BCL-2 or BCL-X_L_. DNA damage also blocks transcription of the JNK phosphatase MKP-1, releasing its inhibitory effect over JNK which in turn induces autophagy. PARP1 activation in response to DNA damage leads to reduction in both NAD^+^ and ATP pools, with the latter causing activation of AMPK and induction of autophagy. The DDR-autophagy axis has major implications for DNA damage repair, senescence, cell survival versus death, cytokine secretion and modulation of the immune response (see text for details). Black arrows (↑) and perpendicular lines (⊥) denote activation and suppression, respectively. Red arrows denote transcriptional regulation.

DNA damage response utilizes proteins involved in sensing, signaling, and repair of DNA damage. While the early activation events that follow DNA breaks are well elucidated, the primary signal which triggers DDR remains incompletely understood. It has, however, been proposed that when a DNA lesion occurs, it is accompanied by relaxation of chromatin through a series of post-translational histone modifications that include poly-(ADP-ribosylation) which is catalyzed by poly (ADP-ribose) polymerases (PARPs), phosphorylation and acetylation (Lukas et al., 2011). These chromatin responses “freeze” transcription and replication around the site of DNA lesion to facilitate subsequent repair. They also provide access to DDR sensors such as the Mre11–Rad50–Nbs1 (MRN) complex which binds double strand breaks (DSBs) and recruits ATM kinase (**Figure 1**) and the replication protein A (RPA) complex which responds predominantly to single strand DNA lesions and recruits ATR kinase. The binding of these kinases to damaged DNA triggers the recruitment of additional proteins, many of which become phosphorylated and activated to further transduce signals that orchestrate DNA replication, cell cycle control, transcription, repair of damage, and/or survival versus death.

Thus, ATM bound to DSBs in conjunction with the MRN complex, undergoes autophosphorylation and activation. In turn, ATM activates various downstream effector proteins (Marechal and Zou, 2013), including Chk2 and Chk1 involved in cell cycle control, the tumor suppressor p53 which regulates cell survival versus death, HDAC1 and HDAC2 which are responsible for chromatin remodeling (Kim et al., 1999), the BASC complex containing DNA damage repair proteins (Wang et al., 2000), the senescence regulator ARF (Velimezi et al., 2013) and transcription factors such as FOXO3 which regulates genes involved in DNA repair (Tran et al., 2002). ATR shares at least some of these ATM functions, for example capacity to phosphorylate p53 and Chk1 in response to irradiation (Liu et al., 2000; Zhao and Piwnica-Worms, 2001).

In addition to the aforementioned phosphorylation targets, ATM forms a nuclear complex with PARP1 and NEMO (also known as IKKγ). Within this complex, NEMO is subjected to a series of post-translational modifications including phosphorylation, SUMOylation and ubiquitination that lead to its nuclear export (Mabb et al., 2006; Wu et al., 2006). Once in the cytoplasm, NEMO orchestrates the formation of a high molecular weight multiprotein complex which includes the kinases TAK1, IKKα, and IKKβ which are responsible for a signaling cascade that leads to translocation of cytoplasmic RelA NF-κB to the nucleus. Thus, ATM links DNA damage to NF-κB activation (**Figure 1**).

TAK1 also engages the JNK pathway which activates various transcription factors, including AP1. NF-κB and AP1 transcribe genes involved in cytokine synthesis and, depending on the duration of activation, cell survival versus death. Moreover, ATM utilizes JNK to coordinate optimal p53 activation. Thus, on one hand, ATM directly phosphorylates p53 at Ser^15^ which reduces its affinity for the ubiquitin ligase HDM2 leading to p53 stabilization. However, ATM also impacts on p53 indirectly through activation of JNK which phosphorylates p53 at Thr^81^ enhancing its transcriptional activity (Buschmann et al., 2001) (**Figure 1**). DNA damage has been reported to activate JNK also through the transcriptional repression of the JNK phosphatase MKP1 caused by transcription-blocking DNA lesions (Hamdi et al., 2005). Further studies are needed to establish the relative contribution of the ATM-NEMO-TAK1 versus MKP1 pathways to JNK activation which is likely to depend on the extent of DNA damage.

A major end-point of DDR is the activation of the DNA damage repair system. Depending on the type of damage and the phase of the cell cycle, different repair mechanisms are utilized to restore DNA integrity (Jackson and Bartek, 2009; Ribezzo et al., 2016). For example, PARP1 is involved in the repair of single strand DNA breaks by recruiting enzymes necessary for base excision repair such as XRCC1, polymerase β and DNA ligase III to the sites of damage (de Murcia et al., 1997). DSBs are mainly repaired by HR, an error-proof process operating predominantly in the S and G2 phases of the cell cycle, and by NHEJ which is operational in all phases of the cell cycle but is error-prone because of the lack of appropriate undamaged DNA template (Lieber et al., 2003). In general, ATM/ATR-mediated DDR signaling regulates repair by: (a) inducing the transcription of DNA-repair genes, (b) modulating DNA-repair protein activity through post-translational modifications such as phosphorylation, acetylation, ubiquitination or SUMOylation, and (c) by recruiting repair factors to the DNA lesion. If the damage cannot be resolved, chronic DDR signaling triggers cell death or senescence (Roos et al., 2016).

In addition to nuclear DNA, mtDNA is subject to damage following exposure to radiation or chemotherapy. Moreover, a by-product of oxidative production of ATP is the generation of reactive oxygen species (ROS) which may damage both mitochondrial and nuclear DNA (Sedelnikova et al., 2010; Cline, 2012). MtDNA lacks histones which makes it even more susceptible to injury than nuclear DNA. To counteract mtDNA damage, mitochondria possess quality control systems that include antioxidant enzymes and a dedicated repair system which appears to be less elaborate and effective than the nuclear DNA damage repair machinery (LeDoux et al., 1992; Cline, 2012).

## Autophagy: General Mechanisms

Autophagy is a cellular ‘self-eating’ degradation process in which proteins or whole organelles are degraded in lysosomes and recycled to meet the anabolic and bioenergetic needs of the cell. As such, it plays a pivotal role in tissue homeostasis and various human pathologies, including cancer, neurodegeneration, autoimmunity and aging, have been associated with deregulated autophagy (Mizushima et al., 2008).

Depending on the mechanism of delivery of the cargo to the lysosomes, three main types of autophagy have been so-far recognized: micro-autophagy that involves the direct delivery of cargo to lysosomes through lysosomal membrane invaginations; CMA which is typified by the lysosomal import of proteins through their interaction with specialized chaperones; and macro-autophagy which is the most widely studied mechanism of autophagy. In macro-autophagy (hereafter termed autophagy), the cargo is sequestered in double membrane vesicles known as autophagosomes, which are progressively formed by the finely interconnected activities of around 15 autophagy-related (ATG) proteins (Rubinsztein et al., 2012; Wirth et al., 2013). Autophagosomes can engulf cytoplasmic material, protein aggregates, organelles including mitochondria (mitophagy), peroxisomes (pexophagy) and lipid droplets (lipophagy), as well as ribosomes (ribophagy) and parts of the nucleus (nucleophagy).

Autophagosome biogenesis entails three steps: initiation, nucleation, and elongation. Initiation requires the activation of a complex containing ULK1, ATG13, the FAK-interacting protein FIP200 and ATG101. Following activation of this ULK complex, additional proteins are recruited to form an initial double membrane structure called the phagophore. This step requires Vps34 class III PI3K that operates within a progressively formed large macromolecular “nucleation” complex involving Beclin-1, ATG14, and Vps15. Subsequent vesicle elongation allows engulfment and sequestration of organelles or bulk cytosolic material. The elongation step is driven by the covalent conjugation of ATG12 to ATG5, supported by ATG7 and ATG10, and is responsible for the end product of a second reaction, the conjugation of phosphatidylethanolamine (PE) to the microtubule-associated protein light chain 3 (LC3). PE-conjugated (“lipidated”) LC3, known as LC3-II, decorates mature autophagosomes which are targeted to lysosomes together with their cargo. The complete process of autophagy from nucleation to degradation is referred to as autophagic flux and reflects the actual ability of autophagosomes to degrade intracellular components. Autophagic flux can be monitored by determining the degradation of specific autophagic substrates such as SQSTM1 (also known as p62) which interacts with LC3-II (Ichimura et al., 2008). SQSTM1/p62 has attracted significant attention also because of its role in facilitating the degradation of Lys^63^-ubiquitinated, p62-sequestered proteins by autophagy (Pankiv et al., 2007) and its ability to interact with several key components of the NF-κB pathway (Moscat and Diaz-Meco, 2012).

Phagophore formation is typically triggered by the interaction of ATG family proteins with two major regulatory complexes, TORC1 and AMPK. Under nutrient-rich conditions, high TORC1 activity prevents ULK1 activation by phosphorylating ULK1 at Ser^757^ and disrupting the interaction between ULK1 and its activating kinase AMPK (**Figure 1**) (Alexander et al., 2010a,b; Kim et al., 2011). Under starvation conditions, AMPK is activated and induces autophagy by phosphorylating ULK1 at Ser^317^ and Ser^777^ and by activating TSC2 thereby inhibiting TORC1 (Ganley et al., 2009; Laplante and Sabatini, 2012). Recent studies identified an additional, AMPK-independent mechanism of TORC1 inhibition that entails Sestrins, the RAG GTPases and a multiprotein complex called GATOR (GTPase-activating protein activity toward RAGs) composed of two complexes, GATOR1 and GATOR2. GATOR1 plays an essential role in switching off TORC1 upon amino acid depletion by functioning as a GAP for the RAGA/B heterodimer, incapacitating it from interacting with TORC1. GATOR2 is required for lysosomal recruitment of TORC1 by amino acids and is a negative regulator for GATOR1 by inhibiting its GAP activity. The interaction of Sestrins with GATOR2 under nutrient starvation liberates GATOR1 from GATOR2-mediated inhibition (**Figure 1**). Released GATOR1 subsequently binds to and inactivates RAGA/B, resulting in TORC1 suppression (Kim et al., 2015). Among various amino acids, cytoplasmic leucine was found to bind to Sestrins with the highest affinity and to disrupt their interaction with GATOR2 leading to TORC1 activation (Wolfson et al., 2016). Arginine, on the other hand, does not utilize Sestrins but a complex called CASTOR to release GATOR1 from GATOR2-mediated inhibition (Chantranupong et al., 2016). Therefore, different amino acid sensors are involved in TORC1 regulation and, by inference, on autophagy activation.

However, it should be noted that in addition to the aforementioned “canonical” pathway of autophagy, alternative mechanisms of autophagosome formation that do not require the hierarchical participation of all ATG proteins have been described and represent an area of intense investigations (Codogno et al., 2012). For example, unlike amino acid starvation, autophagy induced by deprivation of glucose or inhibition of glucose metabolism does not require ULK1 (Cheong et al., 2011). The topoisomerase inhibitor and clinically relevant chemotherapeutic agent etoposide induces non-canonical autophagosome formation that depends on ULK1 and Beclin but not ATG5, ATG7 or LC3-II (Nishida et al., 2009). Moreover, basal autophagy under nutrient rich conditions operates in an ATG5-independent manner and lipidation of LC3 depends on ATG3 following its conjugation to ATG12 (Murrow et al., 2015).

There is, therefore, a remarkable plasticity in autophagy-related pathways which is likely to be influenced by the type of autophagic stimulus and the expression levels of autophagy regulators. For example, the anti-apoptotic proteins BCL-2 and BCL-X_L_ which are over-expressed in certain lymphomas and carcinomas (Eliopoulos et al., 1995), prevent the induction of autophagy by binding to and inhibiting Beclin-1 (Pattingre et al., 2005). PTEN antagonizes the effects of the PI3K/AKT pathway on TORC1 and thus, positively regulates autophagy (Arico et al., 2001). Conversely, inactivation of PTEN which occurs with high frequency in certain tumor types, may suppress autophagic responses through the concomitant constitutive activation of the PI3K/AKT pathway. The nuclear protein HMGB1 can induce autophagy in a cell-intrinsic manner following its translocation to the cytoplasm and interaction with Beclin-1 to facilitate autophagosome formation (Tang et al., 2010b). However, HMGB1 is also released extracellularly during ‘immunogenic’ cell death (Kepp et al., 2014), and functions in conjunction with other DAMP molecules to induce autophagy in neighboring tumor cells (Tang et al., 2010a). MicroRNA expression may also influence the autophagic process by targeting various autophagy pathway components (Zhai et al., 2013).

## DDR Signaling Activates Autophagy

Accumulating evidence suggests that autophagy can be activated by DNA damage (Robert et al., 2011; Orlotti et al., 2012; Eapen and Haber, 2013) through various, albeit not exclusive routes, summarized in **Figure 1**.

As described above, ATM is a major sensor of DSBs induced by genotoxic stress. ATM links DDR to the induction of autophagy by activating AMPK which in turn phosphorylates TSC2 and removes the inhibitory effect of TORC1 on autophagy (Alexander et al., 2010a,b). The aforementioned ATM-AMPK-TSC2-mediated suppression of TORC1 operates in response to oxidative and nitrosative stress (Alexander et al., 2010a; Tripathi et al., 2013), both of which induce DNA damage and may involve mobilization of ATM to the cytoplasm (Alexander et al., 2010a). AMPK can also activate ULK1 to promote autophagosome formation (Kim et al., 2011).

PARP1, a NAD^+^ dependent chromatin-associated enzyme involved in base-excision repair of small adducts such as those induced by alkylating agents and ROS, is another DDR protein involved in autophagy regulation (de Murcia et al., 1997). DNA damage-induced PARP1 activation is associated with a reduction in both the NAD^+^ and the ATP pool. The latter is paralleled by elevated AMP levels that are sensed by AMPK leading to its activation and induction of autophagy (Rodriguez-Vargas et al., 2012). DNA damage may induce autophagy also through JNK. JNK phosphorylates BCL-2 leading to its displacement from the Beclin-1 complex that primes autophagosomal membrane formation (Wei et al., 2008).

Whereas the rapid induction of autophagy is mediated by post-translational modifications such as phosphorylation, ubiquitination, acetylation and lipidation, the regulation of autophagy may also depend on the execution of particular transcriptional and post-transcriptional programs. Indeed, the β1 and β2 subunits of AMPK are transcriptionally regulated by p53 (Feng et al., 2007) and are indirectly activated by p53 through Sestrin1 and Sestrin2 (Budanov and Karin, 2008). The recent discovery of the Sestrin2-GATOR-RAG pathway regulating TORC1 raises the possibility that the effects of p53 on TORC1 may be influenced by leucine availability. Additionally, p53 up-regulates PTEN expression leading to TORC1 inactivation (Stambolic et al., 2001; Feng et al., 2007). Moreover, DAPK, a transcriptional target of p53, triggers autophagy by phosphorylating Beclin-1 on Thr^119^ thereby releasing it from BCL-2 and BCL-X_L_, and by phosphorylating protein kinase D (PKD), both of which result in Vps34 class III PI3K complex activation and autophagy initiation (Zalckvar et al., 2009a,b; Eisenberg-Lerner and Kimchi, 2012). Another relevant transcriptional target of p53 is the gene encoding DRAM, a lysosomal protein that facilitates the end-stage of the autophagic process (Crighton et al., 2006). However, it should be noted that unlike its nuclear counterpart, cytoplasmic p53 has been associated with activation of mTOR and repression of autophagy but the underlying mechanism remains unclear at present (Tasdemir et al., 2008).

P53 is not the only DDR pathway protein that may transcriptionally regulate autophagy components (Pietrocola et al., 2013). For example, tumor protein p63 isoform ΔNp63α which is phosphorylated by ATM in response to genotoxic stress, transactivates various autophagy regulators including ULK1, ATG3, ATG5, Beclin-1, ATG7, and ATG10 (Huang et al., 2012). The same study showed that phosphorylated ΔNp63α also modulates the expression levels of ATG5, Beclin-1, ATG10, ATG12, ATG16L1, and UVRAG indirectly through the up-regulation of miR-181a, miR-519a, miR-374a, and miR-630, underscoring the contribution of post-transcriptional mechanisms to DNA damage-induced autophagy (Zhai et al., 2013). ATM also mediates the phosphorylation and activation of Che-1, a RNA polymerase II-binding protein which acts to increase the transcription of two mTOR inhibitor genes, Redd1 and Deptor (Desantis et al., 2015). Interestingly, Che-1 expression correlates with the progression of multiple myeloma, a malignancy characterized by high autophagy responses (Desantis et al., 2015). Another example of DDR-mediated transcriptional regulation of autophagy is provided by NF-κB which is activated by ATM-emanating signals and reported to transcriptionally upregulate Beclin-1 (Copetti et al., 2009).

As mentioned above, mtDNA is also subject to damage by irradiation, chemotherapy or ROS. If the extent of this damage exceeds the capacity of the mitochondrial quality control mechanisms, a form of autophagy is activated termed ‘mitophagy’ that leads to the lysosomal degradation of the damaged mitochondria. A critical regulator of this pathway is PINK1 which acts as sensor for mitochondrial damage. PINK1 is physiologically imported to the inner mitochondrial membrane; however, in response to stress-induced loss of mitochondrial membrane potential, PINK1 fails to be imported and is instead retained at the outer mitochondrial membrane where it phosphorylates a number of substrates. One of its targets is the ubiquitin ligase Parkin which catalyzes Lys^63^ and Lys^48^-linked ubiquitination of outer mitochondrial membrane proteins. Lys^48^-linked ubiquitination of target proteins results in their proteasomal degradation whereas Lys^63^-linked ubiquitination leads to recruitment of autophagy adaptors such as optineurin (OPT), nuclear dot protein 52 (NDP52) and SQSTM1/p62, which serve as a bridge for the assembly of the ULK1 complex and autophagosome formation (Youle and Narendra, 2011). The entire mitochondrion eventually becomes engulfed in the autophagosome which then fuses with a lysosome. This autophagy pathway ensures a healthy mitochondrial pool which is essential for normal energy metabolism and cellular homeostasis.

An interesting connection between nuclear DDR signaling and induction of mitophagy has been reported (Fang et al., 2014, 2016). It has been shown that nuclear DNA damage repair defects in xeroderma pigmentosum group A (XPA), ataxia-telangiectasia (AT), or Cockayne syndrome (CS) patients lead to defective mitophagy and the accumulation of dysfunctional mitochondria producing damaging levels of ROS (Fang et al., 2014). In this pathway, systemic DNA damage causes hyperactivation of PARP1 accompanied by NAD^+^ depletion and functional impairment of other NAD^+^-dependent enzymes such as Sirtuins (SIRT). Among them, SIRT1 is of particular interest as beyond its role as deacetylase of proteins involved in DNA repair pathways (Luna et al., 2013; Fang et al., 2016), it also deacetylates PGC1α which is a master transcriptional regulator of several mitochondrial biogenesis genes. Fang et al. (2016) found that reduced SIRT1 activity in XPA disease models resulted in diminished expression of the mitochondrial uncoupling protein 2 (UCP2), a transcriptional target of PGC1α responsible for mitochondrial hyperpolarization and increased import, cleavage and removal of PINK1. The *in vivo* relevance of these findings is highlighted by the observation that XPA, CS and AT patients as well as nematode (*Caenorhabditis elegans*) and rodent models of XPA possess dysfunctional mitochondria that contribute to the neurological and other pathologies manifested in these diseases (Scheibye-Knudsen et al., 2012; Valentin-Vega et al., 2012; Fang et al., 2014). Agents that recover NAD^+^ levels may thus represent a novel approach for therapeutic intervention in XPA, CS, and AT disease.

Overall, there is robust evidence that autophagy is activated by DDR pathways at multiple levels, raising the important question of whether autophagy impinges on functional outcomes of DDR.

## Functional Outcomes of the DDR – Autophagy Axis

### DNA Damage Repair

One of the most fascinating functional outcomes of the DDR-autophagy axis is the regulation of DNA damage repair with major implications in genomic stability, aging and aging-related pathologies including cancer. Indeed, there is now significant evidence to suggest that autophagy is required for the function of ‘error-proof’ HR and NER (Liu et al., 2015; Park et al., 2015; Hewitt et al., 2016; Qiang et al., 2016; Wang et al., 2016). Conversely, autophagy-deficient cells rely mostly on the error-prone NHEJ repair process, which may explain the genomic instability observed in experimental mouse models with defective autophagy (Karantza-Wadsworth et al., 2007; Mathew et al., 2007b) and the observation that in human breast, ovarian and prostate cancers, *beclin-1* is monoallelically deleted (Aita et al., 1999).

SQSTM1/p62 emerges as an important mediator of the effects of autophagy on DNA damage repair. This role has been attributed to a nuclear pool of p62 that accumulates upon autophagy blockade and binds RNF168, inhibiting its E3 ubiquitin ligase activity toward histone H2A. The ensued reduction in chromatin ubiquitination hinders the recruitment of DNA repair proteins such as BRCA1, RAD51, and RAP80 to sites of DSBs and impacts on their ability to repair radiation-induced DNA damage (Wang et al., 2016). RAD51 is also regulated by SQSTM1/p62 through filamin A which physiologically responds to DNA damage by recruiting RAD51 to DSBs. Autophagy impairment increases the interaction of p62 with filamin A, causing proteasomal degradation of both filamin A and RAD51 (Hewitt et al., 2016). Therefore, nuclear p62 that accrues from defective autophagy compromises DNA damage repair and genomic integrity. In line with these findings, nuclear levels and co-localization of p62 with DNA damage foci have been reported to increase with aging, underscoring the potential contribution of this pathway to aging and age-related diseases (Hewitt et al., 2016).

Autophagy is also responsible for the degradation of another chromatin component, HP1α. HP1α maintains a condensed chromatin configuration that hinders the formation of RAD51 nucleoprotein filaments at DSBs. The successful completion of HR repair requires access of RAD51 to DSBs which is achieved by RAD6-mediated ubiquitination and autophagy-mediated degradation of HP1α (Chen et al., 2015).

Chk1, a regulator of DNA damage repair by HR, is another recently described target of autophagy. One study showed that loss of (macro)autophagy by ablation of ATG7 leads to Chk1 ubiquitination and proteasomal degradation which in turn impairs DNA damage repair by HR (but not NHEJ) and genomic integrity (Liu et al., 2015). Another study identified Chk1 as target of CMA. CMA is upregulated in response to DNA damage inflicted by irradiation or chemotherapy, leading to lysosomal degradation of Ser^345^-phosphorylated Chk1 (Park et al., 2015). In contrast, Ser^317^-phosphorylated Chk1 which is the preferred substrate of the proteasome-dependent degradation of Chk1 remains unaffected by CMA inhibition, raising the possibility that different autophagy pathways (e.g., CMA vs. macro-autophagy) may target distinct Chk1 pools. Intriguingly, nuclear accumulation of Chk1 that ensues from defective CMA leads to destabilization of the MRN complex involved in the initial processing of DSBs prior to DNA repair by HR (Park et al., 2015). Thus, loss of CMA may facilitate genomic instability through excessive nuclear accumulation of Chk1 and deregulation of the MRN complex.

There is also evidence that autophagy positively regulates NER, the primary mechanism of repair of UV-induced lesions, by modulating the levels of NER-specific damage recognition proteins XPC, UVRAG, and DDB1/DDB2 (Qiang et al., 2016; Yang et al., 2016). UVRAG is of particular relevance as it binds the Beclin-1/Vps34 complex and increases the catalytic activity of Vps34, therefore acting as an inducer of autophagy (Liang et al., 2006). UVRAG is upregulated by radiation (Yin et al., 2011), localizes to photolesions and associates with DDB1 to promote the assembly and activity of the DDB2–DDB1–Cul4A–Roc1 ubiquitin ligase complex, leading to XPC recruitment and NER (Yang et al., 2016). Conversely, autophagy deficiency has been reported to impair the recruitment of DDB1/2 to UVB-induced DNA damage sites (Qiang et al., 2016). Impaired autophagy also leads to both transcriptional suppression of XPC and reduction in UVB-induced XPC ubiquitination, a process critical for DNA damage recognition. The *in vivo* relevance of these observations is underscored by pharmacological studies in mice showing that inhibition of autophagy by the chemical spautin-1 promotes, whereas stimulation of autophagy by rapamycin reduces UVB-induced tumorigenesis (Qiang et al., 2016).

Notwithstanding the impact of autophagic pathways on the turnover of DNA damage repair proteins, autophagy may have a generic role in DNA repair by regulating the supply of ATP, NAD^+^, and dNTPs that are necessary for this process. For example, repair of DSB requires ATP-dependent chromatin remodeling (Bao and Shen, 2007) and the unwinding of DNA by helicases during NER is an ATP-dependent process (de Laat et al., 1999). NAD^+^ is necessary for the function of PARP1 which is involved in base-excision repair (de Murcia et al., 1997). Moreover, a dNTP pool that is required for DNA replication and repair is maintained by autophagy-mediated degradation of ribonucleotide reductase subunits (Kumar et al., 2011). Likewise, autophagy removes nuclear membrane-enclosed chromosome fragments containing damaged DNA called micronuclei (Rello-Varona et al., 2012), a process that may also contribute to maintenance of genomic integrity.

Overall, the reported findings support a prominent role for autophagy in coordinating the execution of DNA damage repair and warrant further studies into how different repair mechanisms are controlled by distinct modes of autophagy and, conversely, how different autophagic pathways interact to finely balance distinct DNA repair systems.

### Senescence

Activation of autophagy contributes to DNA damage-induced senescence. A study by Kang et al. (2015) has recently unveiled a novel role for selective autophagy in linking DDR to the secretion of cytokines, chemokines, growth factors and proteases that collectively formulate the so-called SASP. The regulation of SASP has attracted significant attention as factors secreted by senescent cells establish an inflammatory environment that may foster the initiation and progression of several pathologies, including aging and aging-related diseases. The work by Kang et al. (2015) has implicated the transcription factor GATA4, which is physiologically targeted for degradation by autophagy through interaction with the autophagy adaptor p62, in SASP regulation. Specifically, upon irradiation or oncogene-induced senescence, GATA4 dissociates from p62 and becomes stabilized, causing induction of TRAF3IP2 and interleukin-1α (IL-1α) expression. In turn, TRAF3IP2 and IL-1α activate NF-κB which regulates major SASP components. Interestingly, activation of this GATA4 pathway depends on the DDR kinases ATM and ATR but is independent of p53 and p16^INK4a^. Thus, selective autophagy for GATA4 functions as an anti-senescence mechanism. Conversely, stimuli that induce senescence activate ATM and ATR to block p62-dependent autophagic degradation of GATA4, resulting in NF-κB activation and SASP induction. The *in vivo* relevance of these findings is highlighted by the fact that GATA4 accumulates in tissues of aged mice and humans as well as in various tissues of irradiated mice and may well contribute to the low level inflammation that typifies aging and aging-related pathologies including cancer (Kang et al., 2015). Interestingly, mice lacking p62 also exhibit an accelerated aging phenotype (Kwon et al., 2012).

Autophagy may also regulate SASP post-transcriptionally through spatial coupling to mTOR (Narita et al., 2011). In cells undergoing H-Ras^V 12^-induced senescence, TORC1-positive lysosomes are recruited at the vicinity of the nucleus, resulting in de-inhibition of ULK1 at more distal locations where autophagosomes can form. As autophagosomes mature, they fuse with TORC1-positive lysosomes, giving rise to a novel membrane compartment called ‘TOR-Autophagy Spatial Coupling Compartment’ or TASCC. The flow of amino acids and other metabolites from autophagolysosomes activates TORC1 and also provides basic building blocks for the synthesis of SASP components. Indeed, blocking the localization of TORC1 to the TASCC by knocking-down the RAG GTPases, results in a reduction in the synthesis and secretion of two major SASP components, IL6 and IL8 (Narita et al., 2011).

In addition to SASP, autophagy may regulate senescence in a cell-autonomous manner through degradation of nuclear lamina components. Dou et al showed that in response to oncogenic stress or chemotherapy-induced DNA damage, Lamin B1 is exported from the nucleus together with chromatin domains, interacts with LC3 and undergoes autophagic degradation (Dou et al., 2015). Interestingly, oncogenic stress fails to induce Lamin B1 degradation and senescence when autophagy is impaired. Moreover, Lamin B1 is not processed by autophagy during starvation. Collectively, these data suggest that the activation of autophagy is required but is not sufficient for the establishment of oncogene-induced senescence and underscore the diversity of autophagic responses to different signals. As Lamin B1 anchors proteins that participate in NER (Butin-Israeli et al., 2013) it would be of interest to examine putative links between nucleophagy and DNA damage repair and their impact on senescence.

A key regulator of cellular senescence is ARF (Liontos et al., 2012). Activated oncogenes, oxidative stress and heat shock can induce the expression of both nucleolar and mitochondria-localized ARF (smARF) in a positive or a negative manner (Sideridou et al., 2011; Liontos et al., 2012; Evangelou et al., 2013; Velimezi et al., 2013; Kotsinas et al., 2014; Sherr et al., 2016). When overexpressed, smARF interacts with BCL-X_L_ and decreases its interaction with Beclin-1. As a result, cell lines overexpressing smARF are more sensitive to the induction of autophagy and senescence caused by starvation or hydrogen peroxide (Pimkina et al., 2009). In contrast, expression of nucleolar ARF does not confer a similar effect (Reef and Kimchi, 2008). These observations indicate that smARF may link autophagy to senescence but whether this applies also in response to DNA damage remains to be determined. It would also be of interest to identify targets of smARF-mediated autophagy. In this regard, a recent study has shown that smARF overexpression depolarizes mitochondria and promotes mitophagy in a Parkin/PINK1-dependent manner (Grenier et al., 2014); the relevance of this finding to DNA damage-induced senescence requires additional studies.

Very recently, a revolutionary hybrid histo/immuno-chemical method of a biotin-linked Sudan Black-B analog has been established to assess the senescent status in *in vitro* and *in vivo* biological settings (Evangelou et al., 2016). This method may constitute an invaluable research tool for the study of the interplay between the molecular pathways implicated in autophagy and DNA damage-induced senescence.

### Cell Survival and Resistance to Genotoxic Therapy

Resistance to chemotherapy represents a major clinical problem for the management of cancer patients. The effects of autophagy on cell survival versus death have attracted particular attention in the context of malignancy as they may affect the outcome of DNA-targeted drug treatments.

**FIGURE 2:**
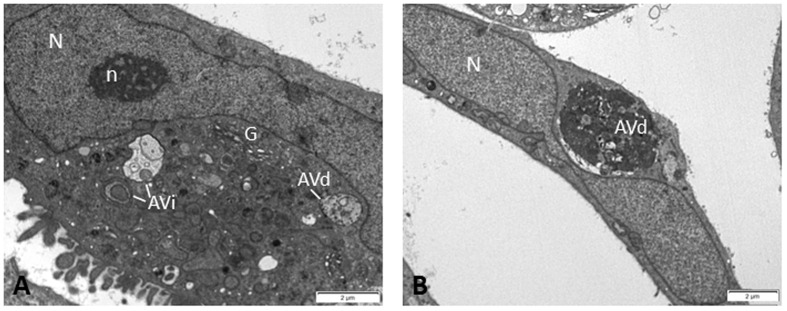
**Electron micrographs of Saos2 p21^WAF1/Cip1^ Tet-ON human osteosarcoma cells after prolonged p21 overexpression (25 days) evading senescence and exhibiting aggressiveness and chemoresistance (Galanos et al., 2016).** In the cytoplasm of these cells, several autophagic vacuoles were observed at different stages of autophagic process, i.e., initial autophagic vacuoles (AVi) **(A)** and degradative autophagic vacuoles (AVd) **(A,B)**, indicating that autophagy may support the chemoresistant features of these cells. Percentage quantitative analysis showed increased number of autophagic vacuoles in Saos2 p21^WAF1/Cip1^ Tet-ON cells after prolonged p21 overexpression (30.1 ± 4.3) compared to control cells (without p21 induction) (3.3 ± 0.7). N, nucleus; n, nucleolus; G, Golgi apparatus.

As discussed above, autophagy intersects with DDR in the regulation of DNA damage repair pathways which are thought to play protective roles in genotoxic cancer therapy. Indeed, the autophagy inhibitor chloroquine has been shown to impair DNA damage repair and to increase the cytotoxic effect of the chemotherapeutic agent carboplatin in breast cancer stem cells (Liang et al., 2016). Accumulating evidence indicates that autophagy is exploited by tumor cells to resist radiation or chemotherapy-induced cell death and that genetic or pharmacological inhibition of autophagy sensitizes malignant cells to genotoxic therapy both *in vitro* and in experimental mouse models (Amaravadi et al., 2007; Pan et al., 2011; Chittaranjan et al., 2014; Wang and Wu, 2014; Filippi-Chiela et al., 2015; Liang et al., 2016; Piya et al., 2016). In line with this is the presence of autophagic vacuoles in Saos2 cells (**Figure 2**), a p53 null human osteosarcoma cell line, which after prolonged expression of p21^WAF1/Cip1^ exhibit enhanced aggressiveness and chemoresistance by deregulating the replication licensing machinery causing replication stress and fuelling genomic instability (Galanos et al., 2016).

These studies have provided the rationale for the clinical application of autophagy inhibitors in combination with established anti-cancer therapies (Huang et al., 2016). Fortuitously, the autophagy inhibitor hydroxychloroquine (a chloroquine derivative) has been used for many years in the management of patients with systemic lupus erythematosus, rheumatoid arthritis and malaria and therefore information about dosage, safety and side-effects has been available. The first phase I clinical trials incorporating hydroxychloroquine to chemo- or radio-therapeutic regimens aim at assessing the tolerability of the combination treatments, as well as dose and schedule optimization and are expected to guide subsequent clinical studies of efficacy (Poklepovic and Gewirtz, 2014).

Whereas as described above, autophagy mediates survival and drug resistance at low DNA damage levels, excessive lesions that cannot be repaired may lead to persistent, unrestraint autophagy which in turn induces a form of cell death termed ‘autophagic cell death’ (ACD). ACD is morphologically distinct from apoptosis and necrosis and is characterized by the sequestration of cytoplasmic materials in autophagosomes (Mathew et al., 2007a; Kroemer et al., 2009). Therefore, depending on the extent of DNA damage, autophagy may play a cytoprotective or cytotoxic role in the determination of cellular fate. The status of the apoptotic versus autophagic machinery components is likely to contribute to this outcome.

### Regulation of Mediators of the Immune/Inflammatory Response

There is significant evidence supporting that DDR and immune response networks functionally interact (Pateras et al., 2015) and that autophagy participates in the regulation of inflammatory pathways (Netea-Maier et al., 2016).

Beyond SASP, autophagy may link DDR to the generation of local and systemic immune responses through the regulation of the so-called ‘immunogenic cell death,’ a form of cell death accompanied by the emission of immunostimulatory DAMPs. These include cell surface-exposed calreticulin and ATP and HMGB1 that are released by dying tumor cells following their exposure to certain genotoxic agents such as ionizing radiation (IR), doxorubicin and oxaliplatin (Kepp et al., 2014). A study by Guido Kroemer’s group has shown that autophagy is critically required for the release of ATP by irradiated tumor cells (Michaud et al., 2011). In turn, ATP stimulates the P2X7 receptor on antigen-presenting cells (APCs) leading to activation of the inflammasome, a large multiprotein signaling complex that regulates the processing of inactive pro-IL-1β to mature, secretable IL-1β (Michaud et al., 2011).

Intriguingly, autophagy also operates in APCs in a cell-intrinsic manner to control secretion of IL-1β and other cytokines. Thus, basal autophagy inhibits IL-1β secretion by degrading both inflammasome proteins (Shi et al., 2012) and pro-IL-1β (Harris et al., 2011). In contrast, under conditions of dual inflammasome and autophagy activation, IL-1β secretion increases through a non-canonical secretory pathway that depends on autophagy components (Dupont et al., 2011). Whereas these studies have not addressed the impact of autophagy on IL-1β biogenesis and secretion in response to genotoxic stress, it is likely that autophagy has a broader role in influencing the extent and duration of inflammation. This may be of particular relevance to and warrants further investigations in aging which is typified in humans by chronic, low-level inflammation, termed ‘inflammaging’ (Franceschi and Campisi, 2014), accumulated genomic damage (Ribezzo et al., 2016) and reduced autophagic activity (Martinez-Lopez et al., 2015).

## Summary and Perspectives

The DDR has been conserved during evolution from bacteria to mammals, serving pivotal cellular functions that include repair of DNA lesions and maintenance of DNA integrity. As prokaryotic cells do not possess lysosomes, autophagy must have originated at a later phase of evolution than the DDR. Indeed, despite some differences in the biochemical pathways involved, autophagy-specific machineries have been identified in all eukaryotes examined, including yeast, plants (*Arabidopsis thaliana*), amoebozoa (*Dictyostelium discoideum*), and metazoa (*Caenorhabditis elegans*, *Drosophila melanogaster*, *Mus musculus, and Homo sapiens*) but not in prokaryotes (Hughes and Rusten, 2007). It has been proposed that the original function of autophagy may have been the adaptation to conditions of starvation through the recycling of intracellular components, and/or an early form of innate immune system allowing the destruction of intracellular bacterial pathogens (Hughes and Rusten, 2007).

However, emerging evidence links autophagy also to the DDR. Herein we have reviewed this evidence and highlighted in particular that: (1) autophagy is activated upon exposure to diverse DNA damaging factors including radiation, chemicals, ROS and oncogenes; (2) the DDR-autophagy link operates in a variety of eukaryotic cell types representing different evolutionary and developmental stages; (3) autophagy is required for several fundamental processes associated with the cellular response to DNA damage such as repair of DNA lesions, senescence, cell death, and cytokine secretion; (4) reduced autophagic flux characterizes human pathologies that are associated with accumulation of DNA damage, including cancer and aging.

Together, these observations raise the possibility that autophagy may have evolved as a quality control system that responds to a wide range of stress conditions including DNA damage, a major cellular stress factor. Unlike the more broadly conserved ubiquitin-proteasome system which regulates the turnover of short-lived proteins in both prokaryotes and eukaryotes, autophagy can target organelles or bulk cytoplasmic and nuclear material for lysosomal degradation. This is a property relevant to DDR in eukaryotic cells that enables them, for example, to deplete mitochondria bearing irreparable DNA damage and to control the number of mitochondria under stress conditions. Notably, the autophagic degradation of dysfunctional mitochondria (mitophagy) operates across the Eukaryota domain, including yeast, nematodes, flies, and mammals (Hirota et al., 2012), contributes to lifespan extension in model organisms (Palikaras et al., 2015) and protects against aging-related pathologies such as cancer and neurodegenerative diseases (Mathew et al., 2007a; Menzies et al., 2015; Fang et al., 2016). Further studies are needed to address the impact of DDR signaling on autophagy-mediated degradation of organelles other than mitochondria and their role in aging and aging-related disease pathogenesis. For example, defects in autophagy-mediated lipid droplet degradation (lipophagy) have been associated with metabolic disease and liver steatosis (Madrigal-Matute and Cuervo, 2016) but whether DDR utilizes this degradation pathway remains largely unexplored. Intriguingly, systemic DNA damage ensued by NER deficiency in mouse adipocytes leads to destruction of white adipose tissue depots (Karakasilioti et al., 2013). Whilst the contribution of autophagic degradation of lipid droplets to this phenotype remains elusive, the aforementioned observation does raise the possibility of putative links between DDR and lipophagy.

The exciting findings that continue to emerge in this field warrant further studies into the complex and often contrasting roles of autophagy in the onset, progression and therapy of various human diseases. This need is underscored by its dual role in malignancy: whereas autophagy prevents genomic instability, a hallmark of cancer, it may promote survival of tumor cells under stress conditions, including those induced by anticancer therapy (Mathew et al., 2007a). As the pathological basis of the vast majority of human diseases is typified by the involvement of multiple cell types and inter-organ communication, further studies are needed to address how tissue-specific deregulation of the DDR-autophagy axis may lead to systemic effects underpinning disease pathogenesis. Along these lines, a recently published study revealed a mechanism by which autophagy in the CNS and periphery coordinate lipophagy in the liver and adipose tissue (Martinez-Lopez et al., 2016). Likewise, the pursuit of autophagic pathways and their functional outcomes will no doubt continue to provide an invaluable side entrance into the complex but fascinating biology of DDR.

## Author Contributions

AE, SH, and VG contributed to the preparation, drafting and final review of the manuscript. SH provided the data depicted in **Figure 2**.

## Conflict of Interest Statement

The authors declare that the research was conducted in the absence of any commercial or financial relationships that could be construed as a potential conflict of interest.

## Abbreviations

AMPK: AMP-kinase
AP1: activator protein 1
APC: antigen-presenting cells
ATM: ataxia-telangiectasia mutated
ATR: ATM- and Rad3-Related
BASC: BRCA1-associated genome surveillance complex
CMA: chaperone-mediated autophagy
Chk1/Chk2: Checkpoint kinases 1/2
DAMP: damage-associated molecular pattern
DAPK: death-associated protein kinase
DDB: damage specific DNA binding proteins 1 and 2
DNA-PK: DNA-dependent protein kinase
DRAM: damage-regulated autophagy modulator
FOXO3: Forkhead box O3
GATA4: GATA Binding Protein 4
HDAC: histone deacetylases
HMGB1: High Mobility Group Box 1
HP1α: heterochromatin protein 1α
HR: homologous recombination
IKKγ: inhibitor of nuclear factor kappa-B kinase subunit gamma
JNK: cJun N-terminal kinase
LC3: microtubule-associated protein light chain 3
MRN: Mre11-Rad50-Nbs1 complex
mtDNA: mitochondrial DNA
NEMO: NF-κB essential modulator
NER: nucleotide excision repair
NHEJ: non-homologous end joining
PI3K: phosphatidylinositol 3-kinase
PINK1: PTEN-induced putative kinase 1
PTEN: Phosphatase and Tensin homolog
RAG: Ras-related GTPases
SASP: senescence-associated secretory phenotype
SQSTM1: sequestosome 1
TAK1: transforming growth factor beta-activated kinase 1
TASCC: TOR-Autophagy Spatial Coupling Compartment
TORC1: mechanistic target of rapamycin complex 1
TRAF3IP2: TNF receptor–associated factor interacting protein 2
TSC2: Tuberous Sclerosis Complex 2
ULK1: unc-51-like autophagy-activating kinase 1
UVRAG: UV radiation resistance-associated gene
XPA: xeroderma pigmentosum group A
XPC: xeroderma pigmentosum, complementation group C
